# LINC01001 Promotes Progression of Crizotinib-Resistant NSCLC by Modulating IGF2BP2/MYC Axis

**DOI:** 10.3389/fphar.2021.759267

**Published:** 2021-09-24

**Authors:** Meiling Zhang, Qian Wang, Zihao Ke, Yijing Liu, Huijin Guo, Shencun Fang, Kaihua Lu

**Affiliations:** ^1^ Department of Oncology, The First Affiliated Hospital of Nanjing Medical University, Nanjing, China; ^2^ Department of Respiratory Medicine, The Affiliated Brain Hospital of Nanjing Medical University, Nanjing, China

**Keywords:** crizotinib-resistance NSCLC, crizotinib, IGF2BP2, LINC01001, MYC

## Abstract

**Background:** Crizotinib is a microtubule-related protein-4-anaplastic lymphoma kinase (EML4-ALK) multi-target tyrosine kinase inhibitor applied in the treatment of ALK-rearranged NSCLC. However, the specific molecular mechanism underlying its therapeutic effect remains unclear. Therefore, the purpose of this research is to explore the mechanism by which crizotinib targets NSCLC with ALK-rearrangement, mainly whether it is related to LINC01001 in regulating NSCLC progression via IGF2BP2/MYC axis.

**Methods:** RT-qPCR is conducted to evaluate the mRNA levels of LINC01001, IGF2BP2 and MYC in A549/R and H1299/R cells. CCK-8 and EdU assays are performed to assess the viability and proliferation of A549/R and H1299/R cells. Western blot is conducted to measure the levels of PCNA and Ki-67 proteins in A549/R and H1299/R cells. FACs and TUNEL are performed to detect apoptosis of A549/R and H1299/R cells. Immunohistochemical staining is performed to assess the levels of Ki67 in crizotinib-resistant NSCLC tissue. Bioinformatics analysis of multiple CLIP (crosslinking-immunoprecipitation) data found potential binding sites between LINC01001 and IGF2BP2, IGF2BP2 and MYC, that are confirmed by RIP assay and RNA pulldown assay.

**Results:** Our findings illustrated that LINC01001 is highly expressed in crizotinib-resistant NSCLC cells and associated with poor overall survival of NSCLC patients. Inhibition of LINC01001 depresses crizotinib resistance of NSCLC cells. LINC01001 interacts with IGF2BP2, and inhibition of IGF2BP2 depresses crizotinib resistance of NSCLC cells. IGF2BP2 interacts with the mRNA of MYC, and LINC01001 overexpression increases crizotinib resistance of NSCLC via MYC.

**Conclusion:** LINC01001 promotes the progression of crizotinib-resistant NSCLC by modulating the IGF2BP2/MYC axis. Our research clarifies the specific mechanism of crizotinib-resistance in NSCLC treatment.

## Introduction

Non-small cell lung cancer (NSCLC), one of the most common malignant tumors, is one of the leading causes of cancer-related death worldwide (Friboulet et al., 2014a). Furthermore, the 5 years survival rate of NSCLC patients is unsatisfactory due to its metastatic trend and continuous recurrence after surgery (Herbst et al., 2018). Therefore, understanding the pathogenesis of NSCLC will contribute to providing a new treatment strategy for the treatment of NSCLC.

Crizotinib is a mesenchymal-epithelial transition (MET)/ALK multi-target tyrosine kinase inhibitor, which entered phase I early clinical studies in 2007 (Camidge et al., 2018). On August 26, 2011, the US Food and Drug Administration (FDA) approved crizotinib to treat NSCLC (Katayama et al., 2011; Heigener and Reck, 2018). Crizotinib has made significant progress in treating NSCLC with ALK-rearrangement, but with its extensive use, tumor cells develop drug resistance and become less and less sensitive to the treatment (Dagogo-Jack and Shaw, 2016; Nakagawa et al., 2020). Thus, crizotinib resistance turns out to be a big challenge in ALK-rearrangement NSCLC treatment (Casaluce et al., 2016; Mok et al., 2020). Therefore, it is urgent to understand the mechanism of drug resistance of crizotinib in NSCLC and seek novel therapeutic strategies.

Long non-coding RNAs (LncRNAs) are vital intracellular regulatory molecules with functional activity in various physiological processes (Dahariya et al., 2019). Researches have shown that LINC01001 is highly expressed in lung adenocarcinoma and is related to the poor prognosis of patients (Li et al., 2016). Therefore, we speculate that LINC01001 may be involved in the regulation of NSCLC progression.

The insulin-like growth factor 2 mRNA-binding protein (IGF2BP) family consists of three members, IGF2BP1-1, IGF2BP1-2 and IGF2BP1-3. They are responsible for targeting mRNA stability and are associated with various targets, such as MYC (Bell et al., 2013; Huang et al., 2018). IGF2BP plays a vital role in tumor cell metabolism, proliferation and differentiation, and is regulated by multiple lncRNAs. For example, LncRNA LINRIS promotes colorectal cancer cell proliferation by stabilizing IGF2BP2 (Wang et al., 2019). In addition, LncRNA 91H regulates the migration and invasion of colorectal cancer cells by interacting with IGF2BP2 (Gao et al., 2020). Therefore, we speculate that LINC01001 may regulate the proliferation and apoptosis of NSCLC cells by regulating the IGF2BP3/MYC axis.

This study aims to investigate whether IGF2BP2/MYC axis is involved in the specific mechanism of LINC01001 in crizotinib-resistant NSCLC progression.

## Materials and Methods

### Patients and Tissue Specimen

NSCLC tissues and matched adjacent non-tumor specimens were collected in the First Affiliated Hospital of Nanjing Medical University from May 2019 to May 2020. The patients were free of diseases such as infectious diseases and other cancers. All samples were histopathologically confirmed, and patients were treated with crizotinib before surgery. All patients were informed before inclusion in the study and submitted their written informed consents. All experimental protocols were approved by the Ethics Committee of the First Affiliated Hospital of Nanjing Medical University and were following the Helsinki Declaration of 1964 and its subsequent amendments.

### Cell Culture

NSCLC cell lines, including A549 and H1299 cells, were obtained from Punuosai Life Technology Co., Ltd (Wuhan, China). Dulbecco’s modified Eagle’s medium (DMEM, Roche, Basel, Switzerland) supplemented with 10% fetal bovine serum (FBS) (Roche, Basel, Switzerland) and 1% penicillin-streptomycin solution (Solarbio, Beijing, China) was applied to cultured cells in a humidified incubator containing 5% CO_2_ at 37°C.

### Establishing Crizotinib-Resistant Non-Small Cell Lung Cancer Cell Model

To establish the crizotinib-resistant NSCLC cell lines, A549 or H1299 cells were cultured in a complete medium with the crizotinib (Sigma, #PZ0191) concentration starting from 100 nM to a final concentration of 1 μM over 6 months. The fresh medium containing drug was changed every 72–96 h.

The established crizotinib-resistant cells were maintained in a complete medium with 0.5 μM crizotinib and applied to cultured cells in a humidified incubator containing 5% CO_2_ at 37°C.

### IC_50_ of Crizotinib on Non-Small Cell Lung Cancer Cell Model Cells

A total of 1 × 103 A549 or H1299 cells were randomly divided into three parts and added into the 96-well plates. Following treatment with crizotinib (Pfizer, Inc., New York City, NY, United States) for 72 h, the cells were incubated with Cell Titer-Glo analysis reagent (Promega, Beijing, China) for 15 min, and the cell viability was determined by Centro LB 960 microplate photoluminescence instrument. GraphPad Prism 5.0 was used to draw the relationship between cell viability percentage and inhibitor concentration. The IC_50_ value and dose response curve were generated by the nonlinear regression of log to the response, and IC_50_ was defined as the concentration that reached half the maximum effect without observing complete killing.

### Cell Transfection

The transfection dose for sh-LINC01001, sh-IGF2BP2, sh-MYC, pcDNA- LINC01001 plasmids and its negative control sh-NC and pcDNA-NC (synthesized by Sangon Biotech, Shanghai, China) were 2 μg for A549/R and H1299/R cells in each well of 6-well plates. All the transfection was performed using Lipofectamine™ 3,000 Transfection Reagent (Takara, Kusatsu, Japan). Following 48 h transfection, A549/R and H1299/R cells were applied to subsequent experiments.

### RT-qPCR

TRIZOL reagent (Takara, Kusatsu, Japan) was applied to extracted total RNA from NSCLC cell lines and the RNA concentration was quantitated by absorbance at 260 nm. M-MLV Reverse transcriptase (RNase H) kit (Takara, Kusatsu, Japan) was used to synthesize cDNA. RT-qPCR was performed as previously described (Dong et al., 2017). Primers applied to this research were shown in Table 1.

**TABLE 1:** Primer sequences.

Primer name	(5′-3′) Primer sequences
F-LINC01001	5′-ATG​CAC​TTG​AGC​AGG​GGT-3′
R-LINC01001	5′-TAG​GAG​CAT​AAT​GTA​GAA-3′
F-IGF2BP2	5′-GTT​CCC​GCA​TCA​TCA​CTC​TTA​T-3′
R-IGF2BP2	5′-GAA​TCT​CGC​CAG​CTG​TTT​GA-3′
F-MYC	5′-CCC​TCC​ACT​CGG​AAG​GAC​TA-3′
R-MYC	5′-GCT​GGT​GCA​TTT​TCG​GTT​GT-3′
F-GAPDH	5′-ATG​GAA​ATC​CCA​TCA​CCA​TCT​T-3′
R-GAPDH	5′-CGC​CCC​ACT​TGA​TTT​TGG-3′

### CCK-8 Assay

Cell Counting Kit-8 (Beyotime, Nanjing, China) was used to assess the viability of A549/R and H1299/R cells. Briefly, A549/R and H1299/R cells that were transfected with corresponding plasmids were inoculated onto 24-well plates for 24 h. Subsequently, 10 μl of CCK-8 solution was added to the cell wells, incubated at 37°C for 2 h, and finally a fluorescent microplate reader was used to detect the light absorbance at 450 nm.

### EdU Assay

A549/R and H1299/R cells that were transfected with corresponding plasmids were inoculated onto 24-well plates for 24 h, then, the EDU media was added. After incubation for 2 h, the culture medium was removed, the cells were digested with trypsin, and then washed twice with 1×PBS. Cells were fixed with formaldehyde for 30 min, decolored with glycine, and washed in PBS 2 times. Subsequently, cells were soaked in 0.5%Triton X-100 for 10 min, and then washed twice with 1×PBS. Finally, the staining was performed using the Cell Light ™EDU Cell Proliferation Assay (Sigma, St. Louis, MO, United States) according to the previously published protocol (Xiao et al., 2019).

### Western Blot

Total proteins were isolated from A549/R and H1299/R cells that were transfected with corresponding plasmids by using cell lysis buffer (Beyotime, Nanjing, China). Western blots were conducted according to previously described (Cui et al., 2019). All antibodies used in this research were obtained from Abcam (Cambridge, United Kingdom, 1:1000), including PCNA and Ki-67proteins. β-actin was used as the internal reference. The optical density of protein bands was quantified by ImageJ software (ImageJ Software Inc. United States).

### FACs Analysis

FACs were performed to evaluate A549/R and H1299/R cells apoptosis. In brief, A549/R and H1299/R cells that were transfected with corresponding plasmids were inoculated onto 96-well plates (3,650; Corning, NY, United States) for 24 h. Heterocyanate fluorin (FITC) and propylene iodide (PI) were added to the cells (5 μl/well) and incubated at 37°C for 2 h. The number of apoptotic cells was counted by flow cytometry. Apoptosis is defined as FITC (+) and PI (+).

### Histology

Tumor tissues from xenografts model were obtained and fixed in 4% oaraformaldehyde. Tissues were cut into 4 μm thickness sections and stained with hematoxylin and eosin for histological analysis. The expression of Ki67 was analyzed by specific immuno-stainings. The TUNEL Assay Kit was purchased from Vanzyme (Nanjing, China). A549/R and H1299/R cells that were transfected with corresponding plasmids were used to assess cell apoptosis according to the instructions of the manufacturer, with minor modifications (Fouladi-Nashta et al., 2005).

### Tumorigenesis Assays

All animal experiments were conducted based on protocols approved by the ethical committee of Animal Experiment Center of Hangzhou Medical College (SCXK (zhe) 2019–0,002). For the subcutaneously implanted tumor assay, 1 × 106 H1299/R cells co-transfected with sh-LINC01001, sh-IGF2BP2 or pcDNA-LINC01001 or mock vector which were purchased from Shanghai GenePharma Co., Ltd. They were resuspended in 100 μl PBS and subcutaneously injected into the left flanks of 4 week-old female BALB/c nude mice (7 mice per group). Afterwards, the nude mice were obtained and raised in the Model Animal Research Center of Nanjing University. Tumor growth was monitored once a week. The nude mice were sacrificed after 15 days, and the tumor specimens were weighed, fixed and then stained by H&E staining for histological analyses. The immunohistochemical (IHC) staining was performed to evaluate the levels of Ki67 in tumor tissues, according to the published protocol (Ding et al., 2003).

### Cytoplasmic and Nuclear Ribonucleic Acid Isolation

Bioinformatics analysis of multiple CLIP (crosslinking-immunprecipitation) data found that there was potential binding site between LINC01001 and IGF2BP2. The nuclear and cytoplasmic RNAs or proteins were extracted by the Fisher BioReagents SurePrep Nuclear or Cytoplasmic RNA Purification Kit (Thermo Fisher Scientific) according to the manufacturer’s instructions. RT-qPCR analysis was performed to assess the localization of LIC01001 as previously reported (Zhang et al., 2018).

### RIP Assay

For the RIP assay, A549/R and H1299/R cells that were transfected with corresponding plasmids were collected and lysed in complete RIP lysis buffer. Subsequently, the cell extract was incubated with RIP buffer containing magnetic beads conjugated to human anti-Ago2 antibody (Millipore, United States). Samples were incubated with proteinase K with shaking to digest proteins, and the immunoprecipitated RNA was purified. Finally, the isolated RNA was subjected to real-time PCR analysis.

### RNA Pulldown Assay

The cell lysates of A549/R and H1299/R cells that were transfected with corresponding plasmids were harvested and incubated with Dynabeads M-280 Streptavidin (Invitrogen, CA, United States of America) at 4°C for 3 h following the manufacturer’s protocol. Subsequently, the beads were rinsed with ice-cold lysis buffer three times and washed once with high-salt buffer (0.1% SDS, 1% Triton X-100, 2°mM EDTA, 20 mM Tris-HCl, pH 8.0 and 500 mM NaCl). The bound RNAs were isolated and purified for subsequent RT-qPCR analysis.

### Statistical Analysis

Data were presented as Mean ± standard deviation (SD) from three independent experiments. GraphPad Prism 5.0 Software (GraphPad Software, Inc.) was used for statistical analysis of all data. *t*-test or one-way analysis of variance was used for comparison between the two groups, and Tukey post-test was used for comparison within multiple groups. When *p* < 0.05, the difference was considered of statistical significance.

## Results

### LINC01001 Is Highly Expressed in Crizotinib-Resistant NSCLC Cells and Associated With Poor Overall Survival of NSCLC Patients

**FIGURE 1:**
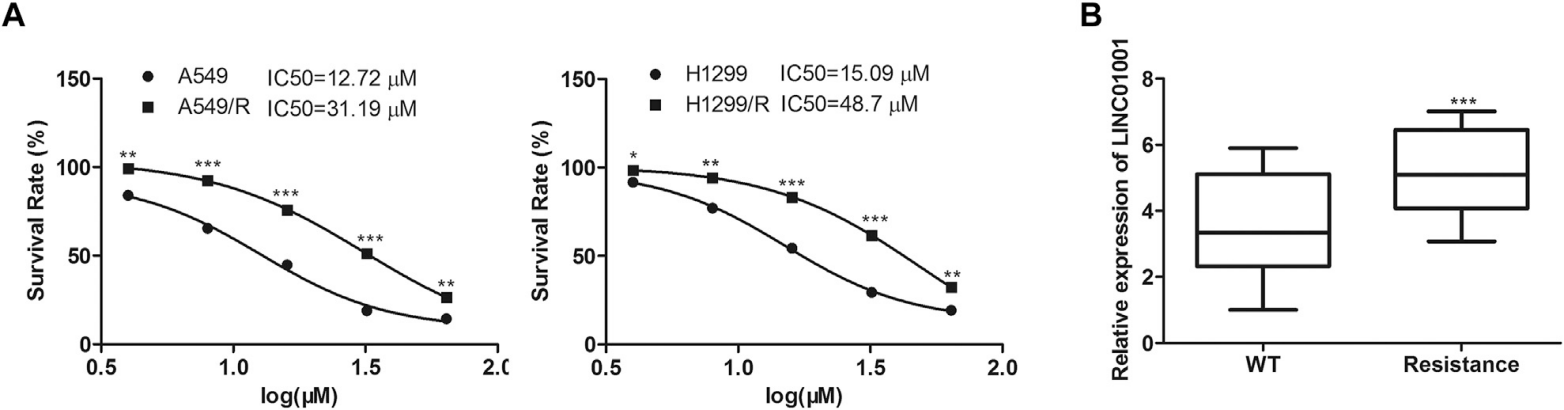
LINC01001 is highly expressed and associated with poor overall survival of NSCLC patients. **(A)** Evaluating the IC50 of crizotinib in NSCLC cells. *p < 0.05, **p < 0.01, ***p < 0.001 vs A549/H1299 group. **(B)** LINC01001 is highly expressed in crizotinib-resistant NSCLC cells. ***p < 0.001 *vs* WT group. Error bars represent SD. Data represent three independent experiments.

The efficacy of crizotinib in the treatment of NSCLC with ALK rearrangement has been confirmed (Friboulet et al., 2014b). We established the crizotinib-resistant NSCLC cell models, and analyzed the sensitivity of crizotinib in A549 cells and H1299 cells. Results showed that the IC_50_ of crizotinib in A549 cells was 10–15 μM, and that in A549/R cells was 30–45 μM. The IC_50_ of crizotinib in H1299 cells was 15–20 μM, and that in H1299/R cells was 40–50 μM (Figure 1A). RT-qPCR analysis indicated that LINC01001 level was significantly increased in the crizotinib-resistant NSCLC cells compared to the parental cells (Figure 1B). These findings revealed that LINC01001 is highly expressed in crizotinib-resistant NSCLC cells and patients.

### Inhibition of LINC01001 Depresses Crizotinib-Resistance of NSCLC Cells

**FIGURE 2:**
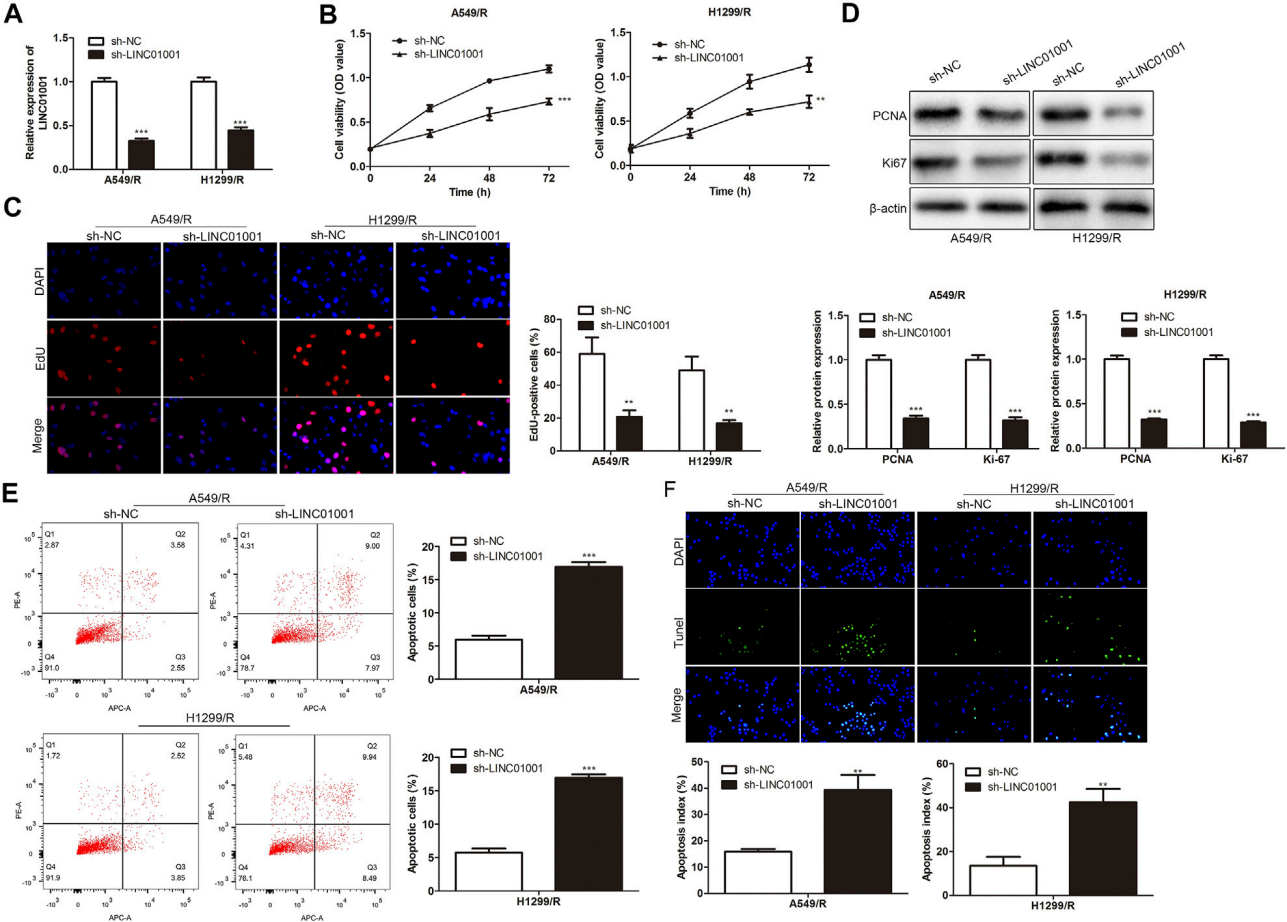
Inhibition of LINC01001 depresses crizotinib-resistance of NSCLC cells. **(A)** RT-qPCR detected the levels of LINC01001. **(B)** CCK-8 assay evaluated the viability of crizotinib-resistant NSCLC cells. **(C)** EdU assay assessed the proliferation of crizotinib-resistant NSCLC cells. **(D)** Western blot measured the levels of proliferation-related proteins, including PCNA and Ki-67. **(E)** FACs assessed apoptosis of crizotinib-resistance NSCLC cells. **(F)** TUNEL staining performed to evaluate apoptosis of crizotinib-resistant NSCLC. **p < 0.01, ***p < 0.001 *vs* sh-NC group. Error bars represent SD. Data represent three independent experiments.

To investigate the regulatory effect of LINC01001 on crizotinib-resistance in NSCLC cells, sh-LINC01001 was transfected into A549/R and H1299/R cells to knockdown LINC01001 expression, sh-NC was used as the negative control for sh-LINC01001. RT-qPCR results indicated that LINC01001 expression was down-regulated by sh-LINC01001, compared with control (Figure 2A). CCK-8 assay analysis showed that LINC01001 knockdown significantly inhibited the viability ofA549/R and H1299/R cells (Figure 2B). EdU assay analysis revealed that LINC01001 knockdown significantly inhibited proliferation of A549/R and H1299/R cells (Figure 2C). Western blot showed that LINC01001 knockdown inhibited the level of cell proliferation-related proteins, including PCNA and Ki-67 (Figure 2D). Furthermore, FACs and TUNEL staining analysis indicated that LINC01001 knockdown significantly accelerated A apoptosis of 549/R and H1299/R cells compared with control (Figures 2E,F). These findings revealed that inhibition of LINC01001 depresses crizotinib-resistance of NSCLC cells.

### Inhibition of LINC01001 Depresses Crizotinib-Resistance of NSCLC *in vivo*

**FIGURE 3:**
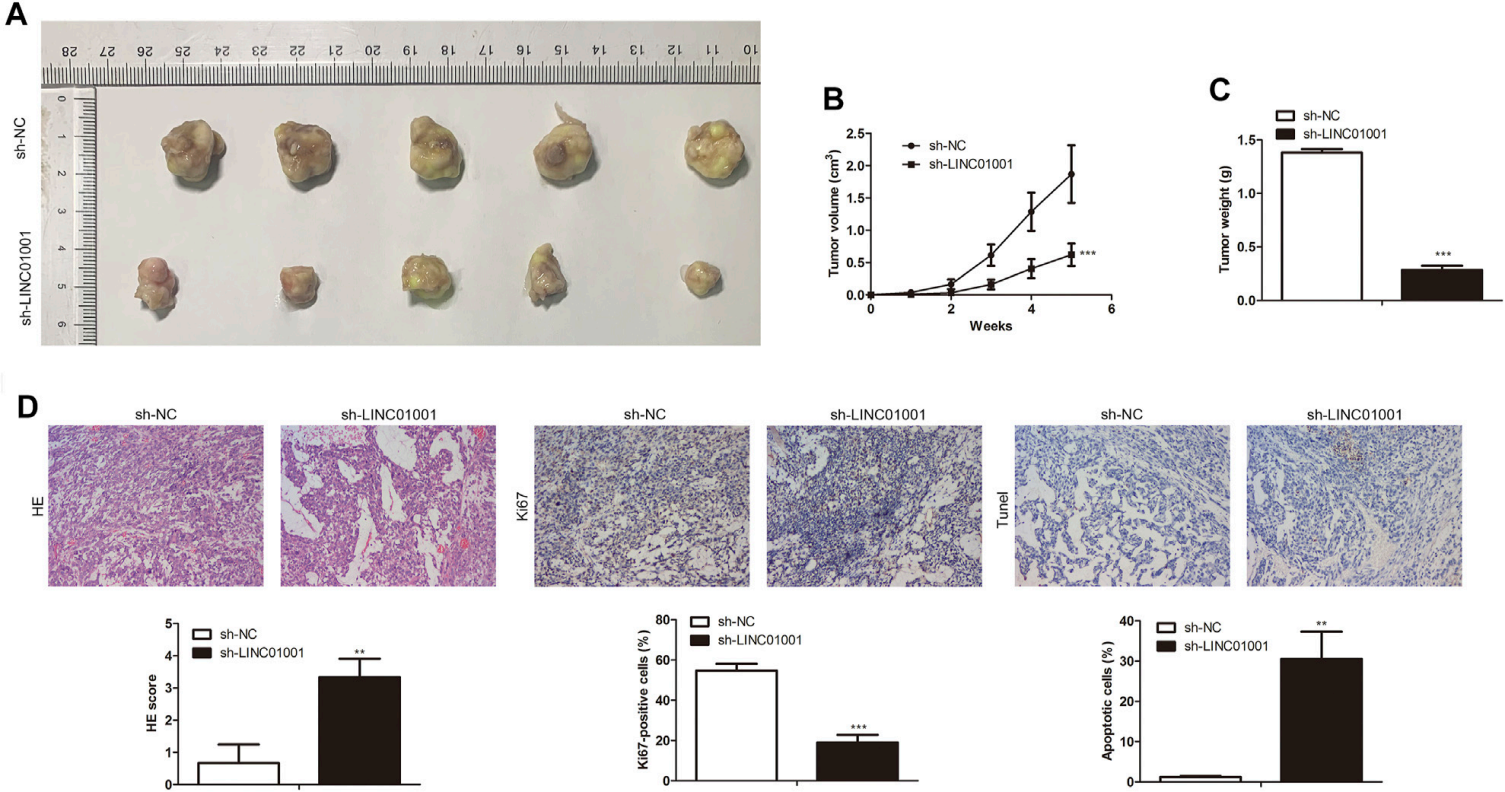
Inhibition of LINC01001 depresses crizotinib-resistance of NSCLC *in vivo*. **(A)** Tumor size. **(B)** Statistical analysis of tumor volume (3 days). **(C)** Statistical analysis of tumor weight (15 days). **(D)** H&E staining and IHC staining assessed the level of Ki67 in tumor tissues (15 days). **p < 0.01, ***p < 0.001 *vs* sh-NC group. Error bars represent SD. Data represent three independent experiments.

Further studies were performed to investigate the roles of LINC01001 in crizotinib-resistant NSCLC tumor growth. sh-LINC01001 was transfected into H1299/R cells to knockdown LINC01001, sh-NC was used as the negative control for sh-LINC01001. The LINC01001 knockdown-H1299/R cells were subcutaneously injected into BALB/C nude mice for tumor formation (*n* = 7). After 7°days, the size and volume of the tumors were analyzed. The results showed that compared with control, LINC01001 knockdown inhibited the volume of the tumors (Figures 3A,B). Furthermore, after 35°days tumors were obtained, statistical analysis of tumors weight showed that, tumor weight in LINC01001 knockdown group was lower than that in control group (Figure 3C). Furthermore, H&E, IHC analysis indicated that Ki67-positive cells in tumor tissues were significantly decreased after transfected with sh-LINC01001. Moreover, TUNEL staining showed that sh-LINC01001 was significantly induced apoptosis. Taken together, compared with control, Tumor growth was inhibited by LINC01001 knockdown in crizotinib-resistance NSCLC tumor tissues (Figure 3D). These findings revealed that inhibition of LINC01001 depresses crizotinib-resistance of NSCLC *in vivo*.

### LINC01001 Interacts With IGF2BP2

**FIGURE 4:**
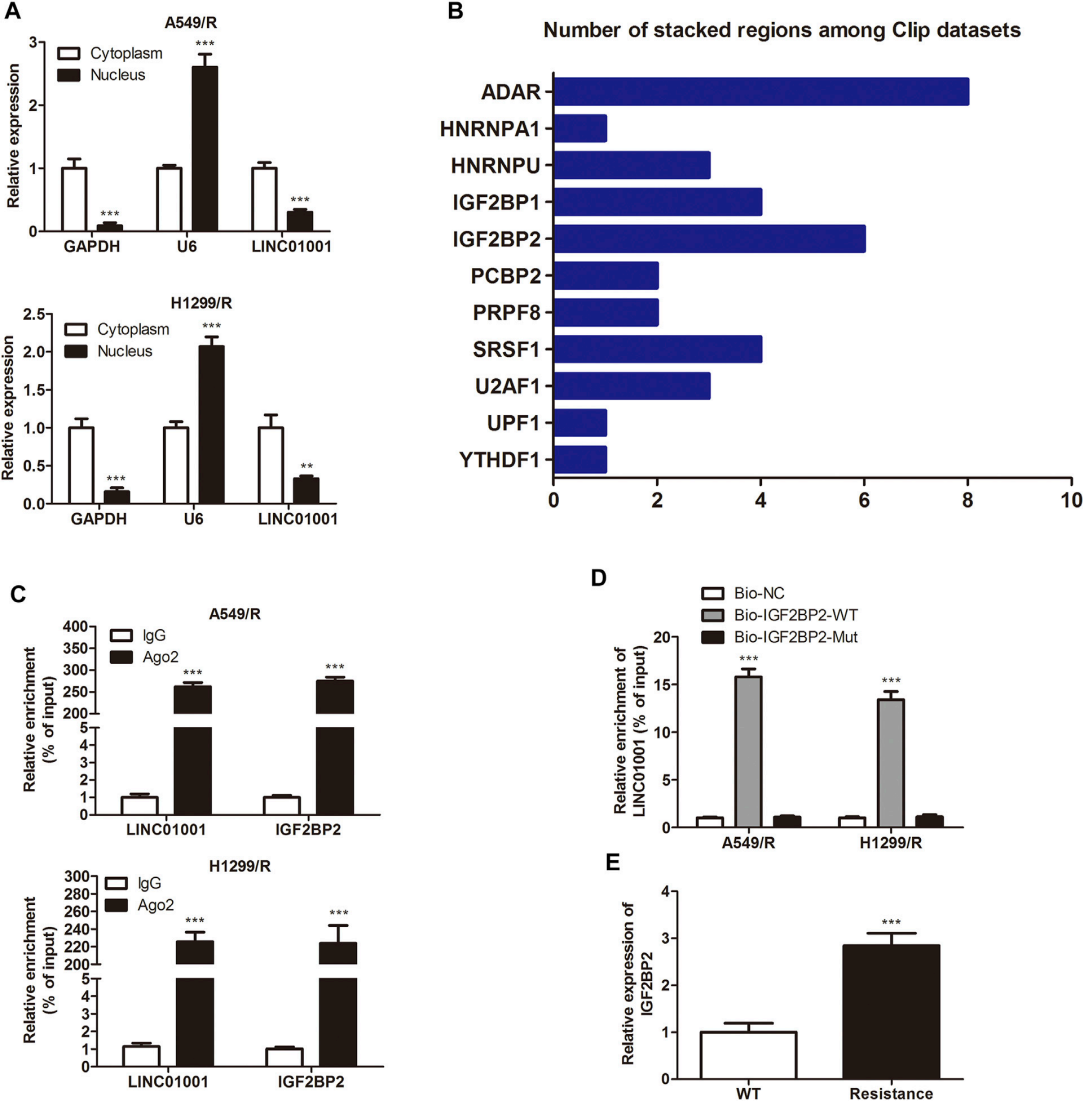
LINC01001 interacts with IGF2BP2. **(A)** Subcellular fractionation evaluated the localization of LINC01001 in crizotinib-resistant NSCLC cells. **p < 0.01, ***p < 0.001 *vs* Cytoplasm **(B)** Bioinformatics analysis indicated LINC01001 interacted with IGF2BP2. **(C)** RIP assay evaluated the interaction between LINC01001 and IGF2BP2 in drug resistant cells. ***p < 0.001 *vs* IgG group **(D)** RNA pulldown assay confirmed the interaction between LINC01001 and IGF2BP2. ***p < 0.001 *vs* Bio-NC group **(E)** RT-qPCR was conducted to assess the level of IGF2BP2 in crizotinib-resistance NSCLC cells. ***p < 0.001 *vs* WT group. Error bars represent SD. Data represent three independent experiments.

LncRNAs are vital intracellular regulatory molecules, that have functional activity in various physiological processes (Li et al., 2020). To confirm the downstream effector of LINC01001 in A549/R and H1299/R cells, subcellular fractionation was performed to evaluate LINC01001 distribution in A549/R and H1299/R cells. The expression of GAPDH and U6 were detected as cytoplasm and nucleus controls. Findings showed that LINC01001 was predominantly localized in the cytoplasm of A549/R and H1299/R cells (Figure 4A). Bioinformatics analysis of multiple CLIP data revealed that LINC01001 interacted with IGF2BP2 (Figure 4B). Furthermore, RIP and pulldown assays was used to varify the interaction between LINC01001 and IGF2BP2 in A549/R and H1299/R cells (Figures 4C,D). The result indicated that biotinylated-IGF2BP2 (Biotin-IGF2BP2-WT) was able to directly precipitate LINC01001, while biotinylated-IGF2BP2 with predicted mutant binding sites (Biotin-IGF2BP2-Mut) could not precipitate LINC01001. Ultimately, RT-qPCR analysis indicated that the levels of IGF2BP2 was significantly up-regulated in A549/R and H1299/R cells, compared to control cells (Figure 4E). These results indicated that LINC01001 interacts with IGF2BP2 in A549/R and H1299/R cells, and IGF2BP2 is highly expressed in A549/R and H1299/R cells.

### Inhibition of IGF2BP2 Depresses Crizotinib-Resistance of NSCLC Cells

**FIGURE 5:**
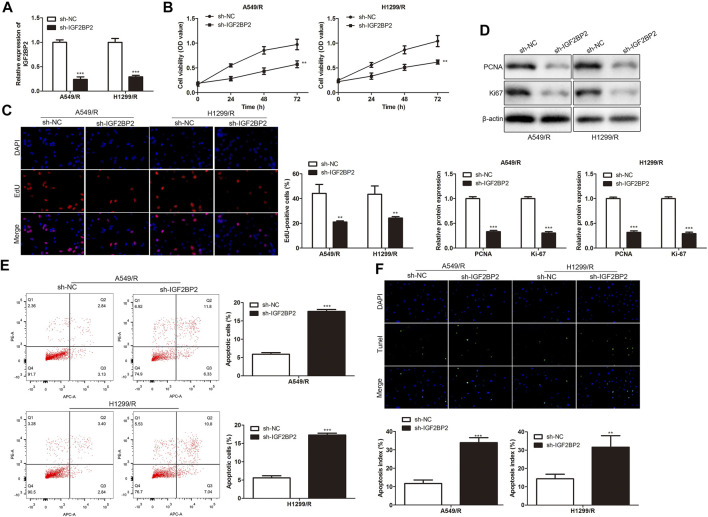
Inhibition of IGF2BP2 depresses crizotinib-resistance of NSCLC cells. **(A)** RT-qPCR detected the level of IGF2BP2. **(B)** CCK-8 assay assessed the viability of crizotinib-resistant NSCLC cells. **(C)** EdU assay evaluated the proliferation of crizotinib-resistant NSCLC cells. **(D)** Western blot measured the level of proliferation-related proteins, including PCNA and Ki-67. **(E)** FACs detected apoptosis of crizotinib-resistant NSCLC cells. **(F)** TUNEL staining assessed apoptosis of crizotinib-resistant NSCLC cells. **p < 0.01, ***p < 0.001 *vs* sh-NC group. Error bars represent SD. Data represent three independent experiments.

To explore the regulatory effect of IGF2BP2 on drug resistance of NSCLC cells, sh-IGF2BP2 was transfected into A549/R and H1299/R cells to knockdown IGF2BP2, sh-NC was used as a negative control for sh-IGF2BP2. RT-qPCR results showed that IGF2BP2 was inhibited by sh-IGF2BP2 plasmids transfection in A549/R and H1299/R cells compared with control (Figure 5A). CCK-8 assay analysis indicated that IGF2BP2 knockdown inhibited the viability of A549/R and H1299/R cells (Figure 5B). EdU assay analysis showed that IGF2BP2 knockdown inhibited proliferation of A549/R and H1299/R cells. Western blot analysis showed significant down-regulation of cell proliferation-related proteins, including PCNA and Ki-67 (Figures 5C,D). Furthermore, FACs and TUNEL staining analysis showed that IGF2BP2 knockdown significantly accelerated apoptosis of A549/R and H1299/R cells, compared with control (Figures 5E,F). These findings revealed that inhibition of IGF2BP2 depresses crizotinib-resistance of NSCLC cells.

### IGF2BP2 Knockdown Decreases Crizotinib-Resistance of NSCLC *in vivo*

**FIGURE 6:**
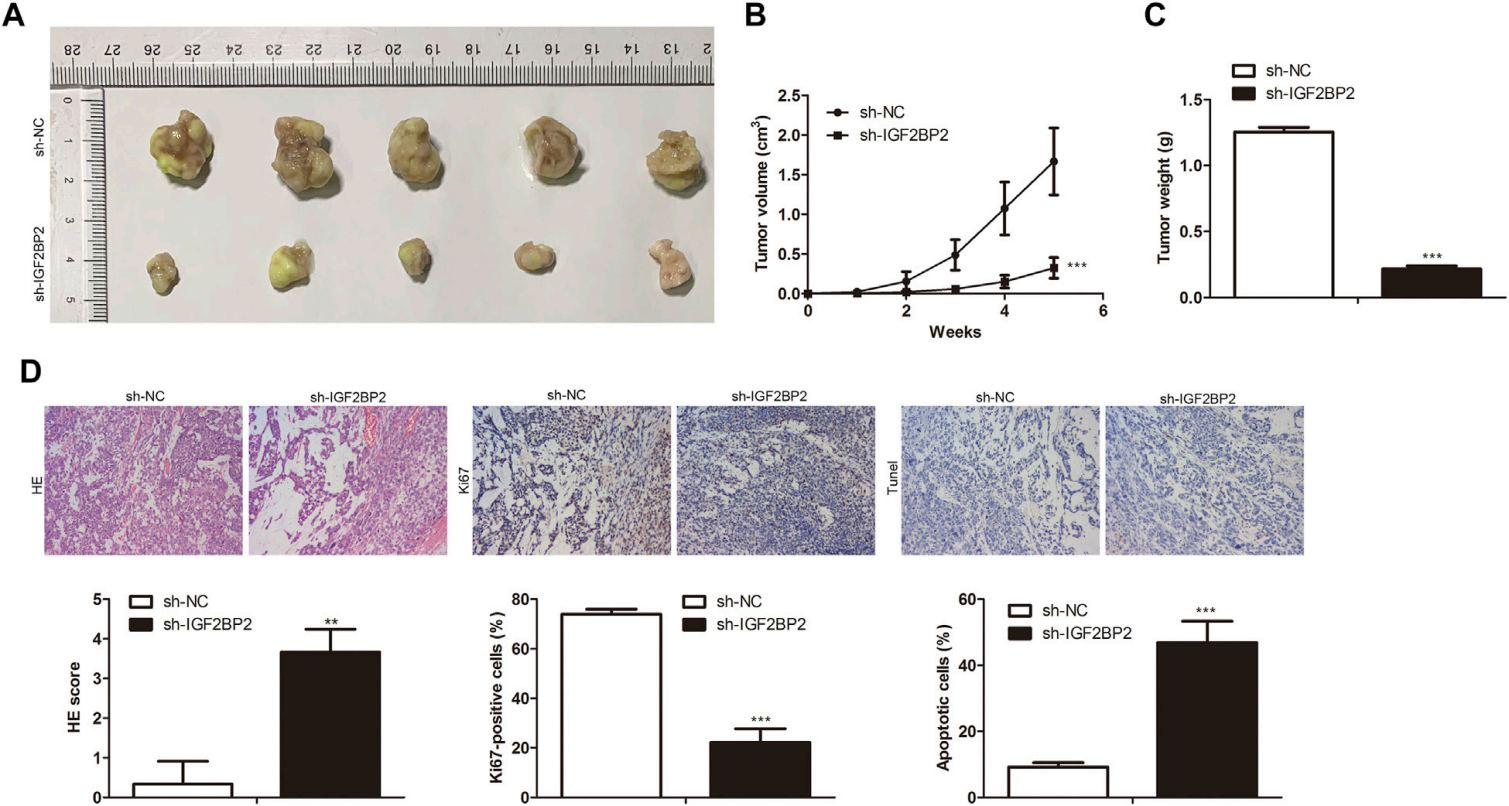
IGF2BP2 knockdown decreases drug resistance of crizotinib-resistant NSCLC ***in vivo***. **(A)** Tumor size. **(B)** Statistical analysis of tumor volume. **(C)** Statistical analysis of tumor weight. **(D)** H&E staining, IHC staining of Ki67 and Tunel assay in tumor tissues. **p < 0.01, ***p < 0.001 *vs* sh-NC group. Error bars represent SD. Data represent three independent experiments.

To investigate the roles of IGF2BP2 in crizotinib-resistant NSCLC tumor growth, sh-IGF2BP2 was transfected into H1299/R cells to knockdown IGF2BP2, and sh-NC was used as the negative control for sh- IGF2BP2. Subsequently, IGF2BP2 knockdown- H1299/R cells were subcutaneously injected into BALB/C nude mice for tumor formation (*n* = 7). After 7°days, IGF2BP2 knockdown inhibited the tumor growth (Figures 6A,B). After 35°days, tumors were collected. It was found that tumor weight in IGF2BP2 knockdown group was lower than that in control group (Figure 6C). Furthermore, IHC staining analysis indicated that the level of Ki67 was lower in IGF2BP2 knockdown crizotinib-resistant NSCLC tumor tissues compared with control tumor tissue, while tunel-positive cells were increased (Figure 6D). These findings revealed that IGF2BP2 knockdown decrease crizotinib-resistance of NSCLC *in vivo*.

### IGF2BP2 Interacts With the mRNA of MYC

**FIGURE 7:**
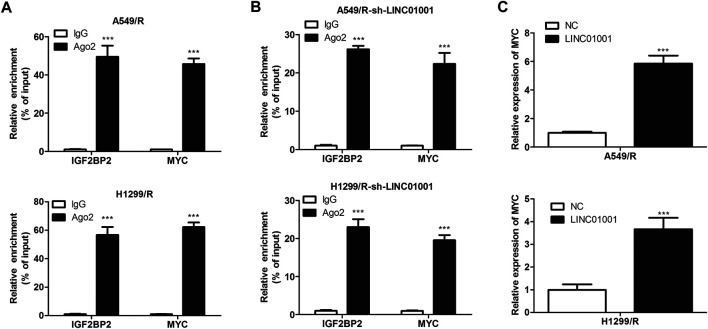
IGF2BP2 interacts with the mRNA of MYC. **(A)** RIP assay evaluated the interaction between IGF2BP2 and MYC mRNA in A549/R cells. **(B)** RIP assay evaluated the interaction between IGF2BP2 and MYC mRNA in LINC01001 knockdown A549/R cells. ***p < 0.001 *vs* IgG group **(C)** RT-qPCR assessed the level of MYC mRNA. ***p < 0.001 *vs* NC group. Error bars represent SD. Data represent three independent experiments.

Further studies were performed to investigate the specific mechanism of LINC01001 in regulating crizotinib-resistant NSCLC progression. RIP assay was performed to verify the interaction between MYC and IGF2BP2. RIP assay revealed the physical interaction between IGF2BP2 and MYC mRNA, where both IGF2BP2 and MYC mRNA were enriched in AGO2 immunoprecipitates in A549/R and H1299/R cells, compared to the control cells (Figure 7A). Subsequently, sh-LINC01001 was transfected into A549/R and H1299/R cells to knockdown LINC01001 expression, sh-NC was used as the negative control for sh-LINC01001. RIP assay was further performed to verify the interaction between MYC and IGF2BP2. Results indicated that no accumulation of IGF2BP2 and MYC was observed in immunoprecipitates of AGO2 (Figure 7B). Furthermore, RT-qPCR analysis showed that LINC01001 knockdown significantly up-regulated MYC level in A549/R and H1299/R cells, compared to the control cells (Figure 7C), indicating that the interaction between IGF2BP2 and mRNA of MYC depends on LINC01001. These results demonstrated that LINC01001 promotes crizotinib-resistant NSCLC progression by modulating IGF2BP2/MYC axis.

### LINC01001 Overexpression Increases Crizotinib-Resistance of NSCLC Cells via MYC

**FIGURE 8:**
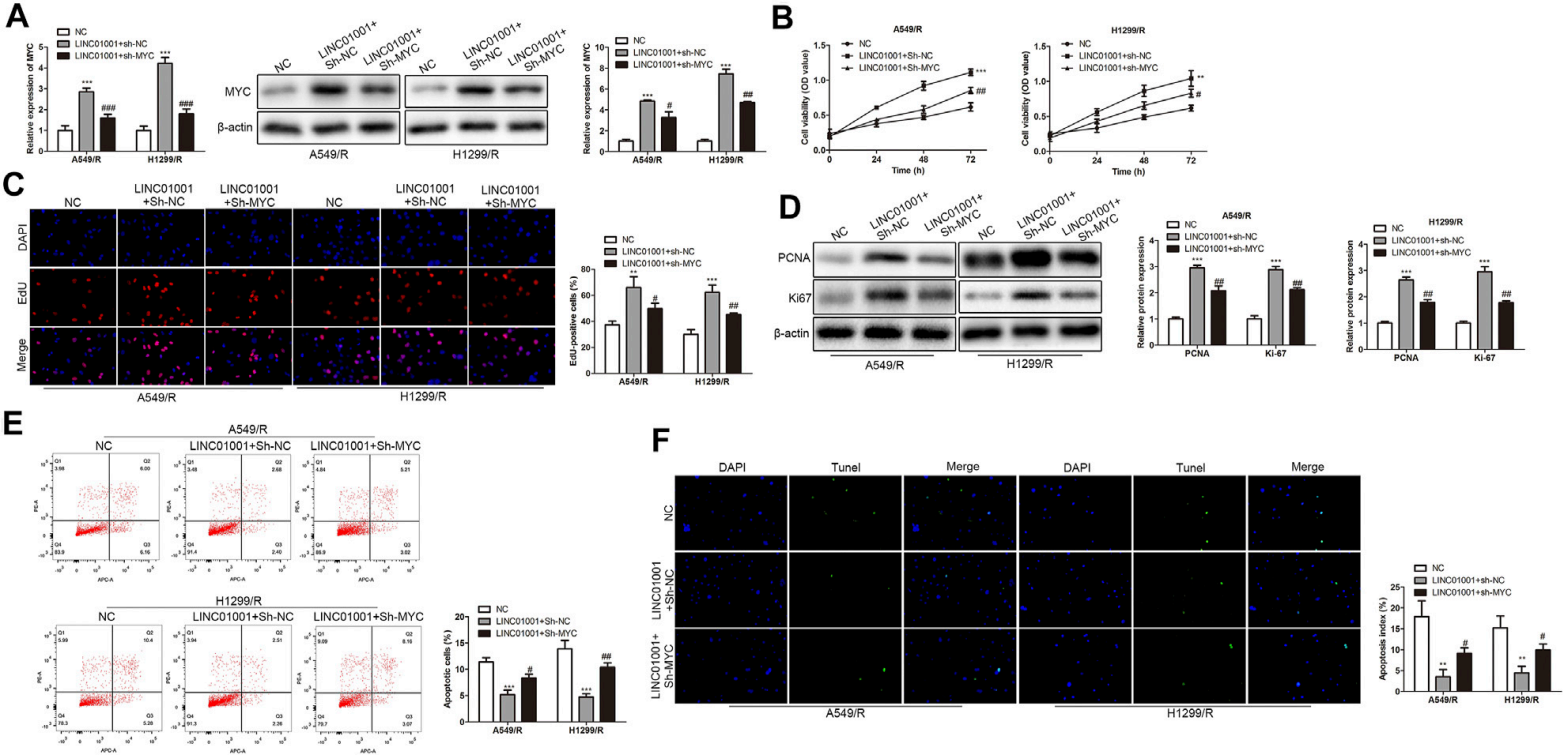
LINC01001 overexpression increases crizotinib-resistance of NSCLC cells via MYC. **(A)** RT-qPCR and western blot detected the level of MYC. **(B)** CCK-8 assay assessed the viability of crizotinib-resistant NSCLC cells. **(C)** EdU assay evaluated the proliferation of crizotinib-resistant NSCLC cells. **(D)** Western blot examined the level of proliferation-related proteins. E, FACs detected apoptosis of crizotinib-resistant NSCLC cells. F, TUNEL assessed apoptosis of crizotinib-resistant NSCLC cells. **p < 0.01, ***p < 0.001 *vs* NC group, #*p* < 0.05, ##*p* < 0.01, ###*p* < 0.001 *vs* LINC01001+sh-NC group. Error bars represent SD. Data represent three independent experiments.

The above experiments proved that there is a negative correlation between LINC01001 and MYC. To further investigate whether LINC01001 regulates crizotinib-resistant NSCLC cells via MYC, pcDNA-LINC01001 and sh-MYC plasmids were co-transfected into A549/R and H1299/R cells for LINC01001 overexpression and MYC knockdown, respectively. pcDNA-NC served as the negative control for pcDNA-LINC01001, and sh-NC served as the negative control for sh-MYC. RT-qPCR analysis showed that compared to the control cells, LINC01001 overexpression significantly up-regulated the levels of MYC in A549/R and H1299/R cells, whereas MYC knockdown promoted the effect (Figure 8A). CCK-8 and EdU assays indicated that compared to the control, LINC01001 overexpression significantly increased the viability and proliferation of A549/R and H1299/R cells, while the effect was alleviated by MYC knockdown (Figures 8B,C). Furthermore, Western blot analysis showed that LINC01001 overexpression up-regulated the expression of cell proliferation-related proteins, including PCNA and Ki-67, whereas MYC knockdown antagonized this effect in A549/R and H1299/R cells (Figure 8D). FACs and TUNEL analysis indicated that the apoptosis of A549/R and H1299/R cells was abolished by LINC01001overexpression, which was alleviated by MYC knockdown (Figure 8 E and 8F). These findings revealed that LINC01001 overexpression increases crizotinib-resistance of NSCLC cells via MYC.

### LINC01001 Overexpression Increases Crizotinib-Resistance of NSCLC via MYC *in vivo*

**FIGURE 9:**
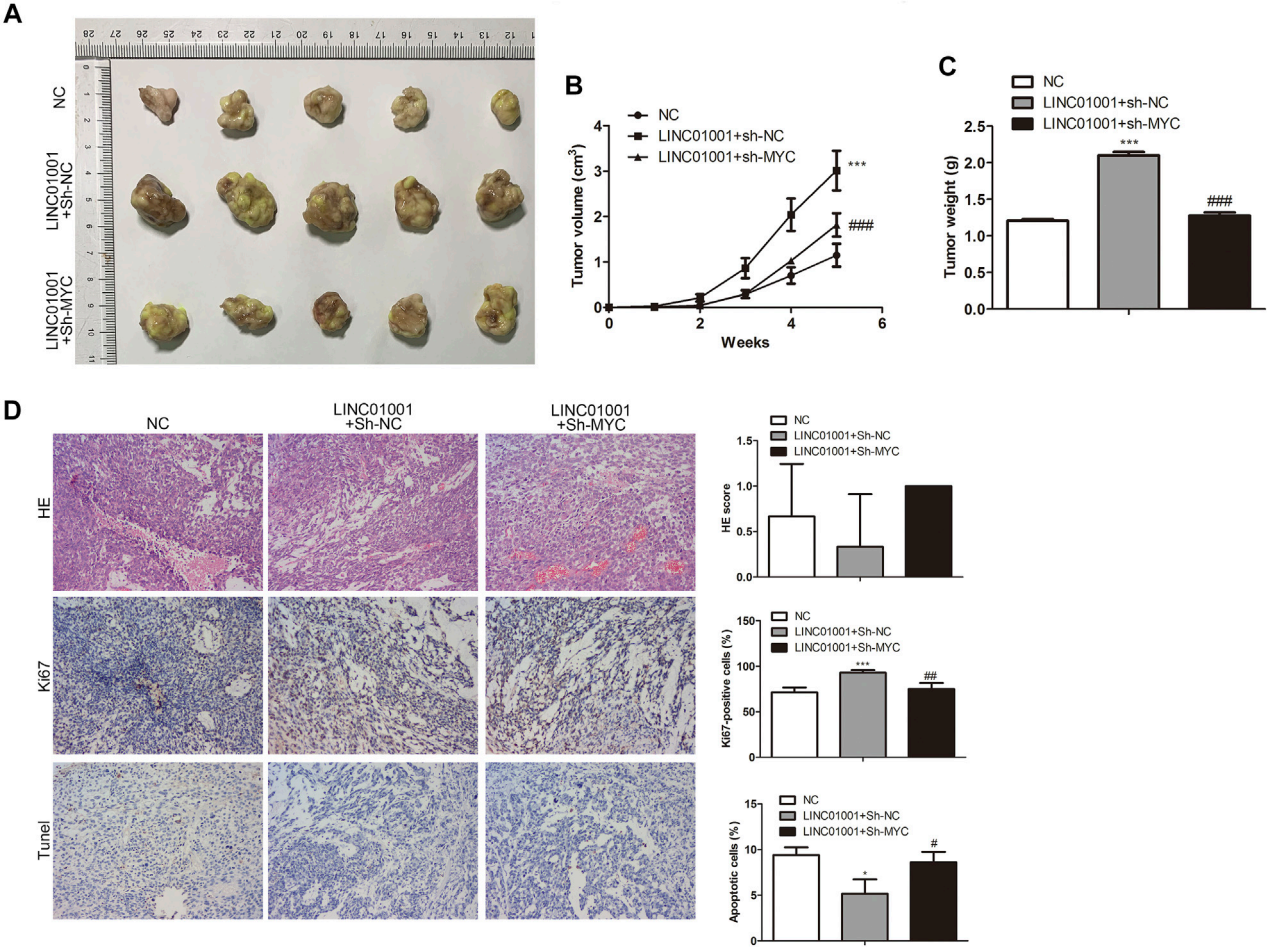
LINC01001 overexpression increases crizotinib-resistance of NSCLC via MYC ***in vivo***. **(A)** Tumor size. **(B)** Statistical analysis of tumor volume. **(C)** Statistical analysis of tumor weight. **(D)** H&E staining and IHC staining of Ki67 in tumor tissues. *p < 0.05, ***p < 0.001 *vs* NC group, #*p* < 0.05, ##*p* < 0.01, ###*p* < 0.001 *vs* LINC01001+sh-NC group. Error bars represent SD. Data represent three independent experiments.

Furthermore, to investigate whether LINC01001 regulates crizotinib-resistant NSCLC tumor growth via MYC, pcDNA-LINC01001 and sh-MYC plasmids were co-transfected into H1299/R cells to overexpress LINC01001 and knockdown MYC, respectively. pcDNA-NC served as the negative control for pcDNA-LINC01001, and sh-NC served as the negative control for sh-MYC. Subsequently, H1299/R cells where LINC01001 was overexpressed and MYC was inhibited were subcutaneously injected into BALB/C nude mice for tumor information (*n* = 7). After 7°days, it was found that tumors were larger in the LINC01001 overexpression group, whereas tumors in MYC knockdown group were smaller compared with control (Figures 9A,B). Subsequently, tumors were obtained following 35°days, statistical analysis of tumors weight indicated that tumor weight in LINC01001 overexpression group was higher than that in control group. However, the effect induced by LINC01001 overexpression was inhibited by MYC knockdown (Figure 9C). Furthermore, IHC staining analysis indicated that the level of Ki67 was up-regulated by LINC01001 overexpression and alleviated by MYC knockdown (Figure 9D). These findings revealed that LINC01001 overexpression increases crizotinib-resistance of NSCLC via MYC *in vivo*.

## Discussion

ALK rearrangement results in abnormal EML4-ALK fusion tumor gene, which activates ALK tyrosine kinase, leading to inhibition of apoptosis and promotion of tumor cell proliferation. Approximately, 3–5% of NSCLC cells possesses ALK rearrangement (O'Bryant et al., 2013). Crizotinib is a potent selective inhibitor of ALK and mesenchymal growth factor tyrosine kinase, which can be applied to the treatment of NSCLC with ALK-rearrangement (Gettinger et al., 2016). However, the therapeutic effect is not ideal due to the emergence of drug resistance. Nowadays, many new generation of ALK inhibitors have been specifically developed and used in clinic, effectively improving the prognosis of patients. However, sequential use of ALK inhibitors can also lead to an accumulation of new ALK mutations in tumor cells. In addition, the safety of these new inhibitors remains to be further verified. Therefore, the new treatment method, which is different from the traditional inhibitor therapy, is particularly important.

In this study, our findings indicate that LINC01001 regulates the progression of crizotinib-resistant NSCLC by modulating IGF2BP2/MYC axis. Our research may clarify the specific mechanism of crizotinib-resistance in NSCLC treatment.

It has been reported that LINC01001 is highly expressed in lung adenocarcinoma and is related to the poor prognosis of patients (Li et al., 2016). Our findings also showed that LINC01001was highly expressed in crizotinib-resistant NSCLC cells and associated with poor overall survival of NSCLC patients, indicating that LINC01001 plays a role in crizotinib-resistance in NSCLC. Further analysis showed that LINC01001 knockdown inhibited viability, proliferation, accelerates apoptosis of crizotinib-resistant A549 and H1299 cells *in vitro*, and inhibits crizotinib-resistant NSCLC tumor growth *in vivo*. These findings indicate that LINC01001 knockdown may impede the progression of crizotinib-resistant NSCLC.

Further investigation showed that there is an interaction between LINC01001 and IGF2BP2, and IGF2BP2 was highly expressed in crizotinib-resistant A549 and H1299 cells. Functional researches showed that IGF2BP2 knockdown inhibited viability, proliferation, accelerates apoptosis of crizotinib-resistant A549 and H1299 cells, and inhibited crizotinib-resistant NSCLC tumor growth, indicating that IGF2BP2 knockdown alleviates crizotinib-resistant NSCLC progression. Furthermore, findings showed that there is an interaction between IGF2BP2 and MYC, which is regulated by LINC01001, suggesting that LINC01001 may regulate MYC via IGF2BP2.

MYC, is one of the most important drivers of tumor development and has been emphasized as a key therapeutic target for cancer treatment of multiple cancer types (Castell et al., 2018). Moreover, MYC is dependent in the treatment of ALK-positive tumors with crizotinib. Higher MYC expression enhances crizotinib chemoresistance via autophagy in anaplastic large cell lymphoma (Shang et al., 2021). Pilling et al. found that silencing of MYC increased sensitivity of ALK inhibitor in lung cancer (Pilling et al., 2018). Overexpression of MYC leading to a reduced sensitivity to crizotinib in NSCC (Rihawi et al., 2019).

Our results furtherly indicated that LINC01001 overexpression accelerates crizotinib-resistant NSCLC cell proliferation, inhibits apoptosis, and accelerates tumor growth via IGF2BP2/MYC axis, indicating LINC01001 regulates crizotinib-resistance NSCLC progression by modulating IGF2BP2/MYC axis.

In summary, our findings illustrated that LINC01001 was highly expressed in crizotinib-resistant NSCLC cells and associated with poor overall survival of NSCLC patients. Inhibition of LINC01001 depressed crizotinib-resistance of NSCLC. LINC01001 interacted with IGF2BP2, inhibition of IGF2BP2 depressed crizotinib-resistance of NSCLC. IGF2BP2 interacted with the mRNA of MYC and LINC01001 overexpression increased crizotinib-resistance of NSCLC via MYC. Therefore, LINC01001 promotes progression of crizotinib-resistant NSCLC by modulating IGF2BP2/MYC axis. The results of this study clarify the mechanism of crizotinib-resistance in NSCLC treatment.

## Data Availability

The original contributions presented in the study are included in the article/Supplementary Material, further inquiries can be directed to the corresponding authors.
